# An 8-Year Breeding Program for Asian Seabass *Lates calcarifer*: Genetic Evaluation, Experiences, and Challenges

**DOI:** 10.3389/fgene.2018.00191

**Published:** 2018-05-29

**Authors:** Pham Van Khang, Truong Ha Phuong, Nguyen Khac Dat, Wayne Knibb, Nguyen Hong Nguyen

**Affiliations:** ^1^GenCology Research Centre, University of the Sunshine Coast, Maroochydore, QLD, Australia; ^2^Department of Science, Technology and Environment, Ministry of Agriculture and Rural Development, Hanoi, Vietnam; ^3^Research Institute for Aquaculture, Nha Trang, Vietnam

**Keywords:** genetic improvement, selection, Barramundi, heritability, correlation and genotype by environment interaction

## Abstract

Selective breeding for marine finfish is challenging due to difficulties in reproduction, larval rearing, and on-growth in captive environments. The farming of Asian seabass (*Lates calcarifer*) has all these problems and our knowledge of the quantitative genetic information (heritability and correlations) of traits necessary for commercial exploitation is poor. The present study was conducted to address this knowledge gap and to provide information that can be applied to sea bass and other aquaculture species. We carried out a comprehensive genetic evaluation for three traits (body weight, total length, and survival) collected from a breeding population for Asian seabass over an eight-year period from 2010 to 2017. Statistical analysis was carried out on 4,567 adult fish at 105, 180, 270, 360, 450, and 570 days post-hatch (dph). The heritabilities (h^2^) estimated for body weight and length using linear mixed model were moderate to high (0.12 to 0.78 and 0.41 to 0.85, respectively) and they differed between the measurement periods. Survival during grow-out phase was analyzed using threshold logistic and probit models. The heritability estimates for survival rate on the underlying liability scale ($h_{L}^{2}$) varied from 0.05 to 0.21. When the observed heritability obtained from the linear mixed model was back-transformed to the liability scale, they were similar but not significant. In addition, we examined effects of genotype by environment (G × E) interaction on body traits. The genetic correlation for body weight between tank and sea cage cultures were high (0.91–0.94) in the first and second rearing periods (180 and 270 dph) but the correlation was decreased to 0.59 ± 0.33 at 360 dph. This suggests that the genotype by environment interaction is important for body traits in this population. Furthermore, the genetic correlations of body weights between different measurement periods were moderate but different from one. This suggests that body weights measured at different time points may be different traits and selection for improved early weight may not capture all genetic expressions in subsequent rearing periods in Asian seabass. Selection of the nucleus in sea cages may produce genotypes that do not perform equally well in tanks, although this deserves further studies to determine a suitable selection environment and optimize the breeding program. This paper discusses challenges encountered during implementation of the selection program for *L. calcarifer*.

## Introduction

Aquaculture of Asian seabass or barramundi (*Lates calcarifer*, Bloch) has been growing in South East Asia and Australia. Regional production of this species in major producing countries increased 350% from ~20,000 tons in 1998 to 90,000 tons in 2017 (FAO Statistics). Asian seabass is highly fecund, euryhaline, and it has a rapid growth rate in both fresh and salt water. In addition, barramundi has good market acceptance and high economic values in many countries (Lawley, 2010; Robinson et al., 2010). Induced spawning is known to be difficult in this species and mass spawning (i.e., keeping broodfish in the same tank with a mating ratio of one female to one or two males) is normally practiced in commercial hatcheries (Evelyn et al., 2013). This practice does not allow the retention of progeny pedigrees unless DNA markers are used for parentage assignment and thus under commercial settings where genetic analysis is not practiced, the contribution made by individual broodstock to future generation is unknown. As a consequence, genetic variation in hatchery populations has declined and this has led to a severe loss in genetic diversity in many cultured stocks (Frost et al., 2006; Loughnan et al., 2015).

Selective breeding is a powerful tool to increase commercial production, and the success of selective breeding programs depends on a systematic approach involving several steps from the establishment of a base population to the development of the breeding objectives and selection strategies (Nguyen, 2016). In aquaculture, quantitative genetics, and selective breeding are in the early stages of development but substantial productivity improvement has already been achieved in fish and shellfish (Gjedrem and Rye, 2016). The principal breeding goals for aquaculture species are growth rate, disease resistance and product quality. The first selection experiments were conducted as long ago as 1919 (Embody and Hayford, 1925) to increase survival to furunculosis (due to *Aeromonas salmonicida)* in brook trout (Salvelinus fontinalis). Since then, several selection programs with the aim of improving growth rate and disease resistance have been reported for a range of fish (Knibb et al., 2016), crustacean (Hung et al., 2013), and mollusc (In et al., 2017). Improvement in growth rates of 5–15% (average 10%) per generation have been achieved—depending on species and testing environments (Gjedrem et al., 2012). It is estimated that in 2010 ~8.2% of global aquaculture production was based on genetically improved stocks. A typical example is Atlantic salmon (*Salmo salar*) where 97% production in Norway has been using improved stocks from artificial selection (Gjedrem, 2012). Hence, there is a strong need to develop an improved genetic line for Asian seabass in order to meet the growing demand for high quality seeds and breeding stock in aquaculture.

In any selective breeding programme, estimation of genetic parameters (heritability and correlations) is needed to understand the genetic basis of quantitative traits (Falconer and Mackay, 1996). They are also required for the evaluation of breeding candidates in order to estimate their genetic merit. These estimates are affected by several factors including genetic resource, sample size, culturing conditions, and the number of generations in experiments. The heritability has been reported for a range of traits in commercial aquaculture species, such as resistance to the bacterial disease columnaris in Rainbow trout (Evenhuis et al., 2015); growth related traits in tilapia (Charo-Karisa et al., 2006; Trọng et al., 2013), common carp (Vandeputte et al., 2004), and barramundi (Wang et al., 2008b; Domingos et al., 2013; Ye et al., 2017). To date, there has been limited genetic parameter estimates in Asian seabass and the published information that exists relates only to body traits recorded in the early stage of growth development, such as at 62 dph (Domingos et al., 2013), and 90 and 270 dph (Ye et al., 2017). Wang et al. (2008b) also analyzed the correlation of body weights between 90 and 270 dph while Ye et al. (2017) reported moderate heritability for growth traits at those measurement periods. Generally, the heritability and genetic correlations for body traits during growth trajectory from tagging to sexual maturity (about 3 kg) are rare in the literature.

The inheritance of survival characteristics during the grow-out phase has not been documented in Asian seabass, although it is one of the two traits (along with growth) that determines economic return for aquaculture enterprises. In contrast, numerous studies have showed existence of the additive genetic components for survival in tilapia (Luan et al., 2008; Thodesen et al., 2013; Ninh et al., 2014; Thoa et al., 2015), salmonids (Standal and Gjerde, 1987; Rye et al., 1990; Vehviläinen et al., 2010, 2012), shrimp (Vu et al., 2017), and molluscs (Dégremont et al., 2005; Liu et al., 2015). The results suggest that these species will respond positively to selection for survival. While systematic environmental effects and management options have been extensively investigated to improve survival rate in *L. calcarifer*, there are no reports of heritability for this trait especially survival rates during the grow-out phase in sea cages. Our study, for the first time, reports quantitative genetic basis of survival of Asian seabass *(L. calcarifer)*.

Another important factor that merits a thorough evaluation in selective breeding programmes is the relationship between genotype and environmental interaction (G × E). The G × E effect is important when the selection and commercial production systems differ so as to develop a genetic line that can tolerate a range of environmental conditions. Understanding the G × E effect on traits of economic importance would aid in the design and optimization of a broadly applicable selective breeding programme for Asian seabass. It has been shown in red tilapia that selection response of genotypes to diverse environmental conditions can vary (Nguyen et al., 2017). Culture systems for Asian seabass are diverse and may involve different water sources (fresh or saline water) or different rearing systems (ponds, tanks, and floating sea cages). Studies in other species, such as Atlantic cod (Kolstad et al., 2006), rainbow trout (Kause et al., 2003), European seabass (Dupont-Nivet et al., 2008, 2010), common carp, and Pacific oysters (In et al., 2017) report low G × E effects. In Asian seabass, Domingos et al. (2013) did not find significant G × E interactions for early growth traits (62 dph) when comparing tank and pond or fresh and sea water environments. However, there is a lack of information about the G × E interactions for *L. calcarifer* grown in tanks and sea cages.

The current study, therefore, aimed to address two major issues: (i) the lack of genetic parameters for body traits and survival during growth trajectory of Asian seabass and (ii) the G × E interaction between tank and sea cage. The study used data collected from the breeding program for *L. calcarifer* at the Research Institute for Aquaculture No III (RIA 3), Vietnam between 2010 and 2017. The founder stocks used to form the base population for selection were collected in 2010 and 2011 from four wild and four hatcheries in the north, central and southern Vietnam. To obtain preliminary information about genetic diversity of these populations, seven microsatellite markers: Lca287, Lca371, Lca154, Lca178, LcaE22, Lca234, and Lca148 (Wang et al., 2008a) were used to analyse polymorphisms of 329 samples including eight wild and domesticated Asian seabass stocks (Phuong et al., 2013). Subsequent generations were produced in 2013, 2015, and 2017. In these years, siblings of each family were cultured in both/either sea cages and/or tanks over a period from 105 to 570 dph. A total of 4,567 fish from 128 full- and half-sib families were performance tested and morphometric measurements for body weight and total length were collected. A full pedigree traced back to the base population was used to estimate genetic parameters for growth traits in *L. calcarifer*. Our results showed that there is potential for genetic improvement to increase both growth and survival in the present Asian seabass population. However, there are challenges that need to be addressed in order to improve the effectiveness of future breeding program for this species.

## Materials and methods

### Ethical statement

All the methods and experimental protocols of this study were performed in accordance with guidelines and regulations approved by the animal ethics committee of the University of the Sunshine Coast, Australia (approval number ANE1613).

### Experimental location

The breeding program for Asian seabass, including breeding, rearing and grow-out experiments, was conducted at the National Mariculture Research and Development Center (MRDC) and Center for Environment and Disease Monitoring in Aquaculture (CEDMA), associated with the Research Institute for Aquaculture No.3 (RIA3) in Khanh Hoa, Central Vietnam (latitude 12.25, longitude 109.19 and it is situated at elevation 10 meters above sea level).

### Origin of the founder stocks

The founder stock comprised four wild and four hatchery fish populations collected from different geographical regions in Vietnam: North (Hai Phong), Central (Khanh Hoa) and South (Vung Tau and Kien Giang) (Supplementary Table S1). They were collected in 2010 and transferred and reared under a common culture environment at the National Mariculture Research and Development Center in Nha Trang.

The fish were initially cultured in 20 m^3^ hatchery tanks and tagged before stocking in sea cages. Tagged fish were assigned randomly to 24 seacages (4 × 4 × 2.5 m) with equal representation of populations in each cage (20 fish per population per cage). After 17 months of culture in seacages, body measurements were made and the growth performance of the eight different populations were evaluated. Regardless of collection locations, wild populations had significant faster growth than those from hatcheries (Supplementary Table S2). The inferior performance of domestictaed stocks was likely resulted from poor management practices of hatcheries that may have caused inbreeding issues. The survival was high (85%) in all stock. There was a significant difference in polymorphism between the wild and domesticated populations. The allele numbers in each locus varied from 11 to 21 alleles/locus in the wild and from 3 to 8 alleles/locus in domesticated populations. The observed level of heterozygosity was also greater in the wild than hatchery sstocks (0.77–0.94 vs. 0.31–0.59). However, there was no clear distinction among populations, Fst value close to zero (Phuong et al., 2013). Based on these results (allele number >6 and observed heterozygosity > 0.6) together with the phenotypic performance, superior animals (400 fastest growth) from the four best stocks (Wild Vung Tau, Wild Khanh Hoa, hatchery fish and wild fish from Kien Giang) were used to form the base population.

### Base population

Parental fish were checked fornightly to confirm that they had reached sexual maturation. Mature broodstock were transferred from seacage to the breeding hatchery and were kept in 20 m^3^ tanks.

In 2011, a partial diallel cross involving fish from the four populations: Vung Tau and Khanh Hoa (both originating from the wild) and hatchery and wild fish from Kien Giang was carried out, following a single pair mating design. Thirty families (one male mated to one female) was produced after 15–20 days. Parental fish were injected using LHRHa and kept in tanks to allow natural spawning. Fish larvea were nursed until fingerling size (28.2 ± 1.2 g, L = 15.3 ± 0.2 cm). A random sample of 30 fish per family was tagged by using Passive Integrated Transponders (PIT). After tagging, the fingerlings were conditioned in tanks for another week before they were transferred into the seacage for grow-out over a period of 17 months. At harvest, body trait data were recorded and genetic evaluation was conducted to estimate breeding values (EBVs) for all individual fish in the pedigree. Based on EBV ranking and relationship of indviduals in the pedigree, the best performing (highest EBVs) individuals were selected to form the base population to produce subsequent generations for selection.

### Family production in subsequent generations in 2014/15 and 2017

In late 2014 and early 2015 the first generation (G1) were produced, involving 45 males and 45 female broodstocks. Mating was based not only on the EBVs but also the genetic relationships with other individuals in the pedigree. Basically, the selection and mate allocation involved three main steps: (1) We ordered families and individuals within each family on genetic merit, (2) Selected best male from best family and assigned to it the best female from best family, and (3) Checked for inbreeding (F) in potential progeny; if F = zero, proceeded to the mating, if not, assigned to best female from second best family; checked for F, and so on. Closely related matings, including full- and half-sibs, were not permitted.

The brood fish chosen for maturation assessment were healthy and normal in body shape. When assessing a prospective female, a catheter with diameter of 1.2 mm and 30 cm long was inserted into the genital pore about 5–10 cm and eggs were taken for microscopic examination. Females were considered ready to spawn when eggs showed uniform size (diameter 0.4–0.5 mm), were separated from each other, and were yellow in color. To assess maturity of males, gentle handling of the fish and slight pressure were applied on their abdomen near the genital pore. This resulted in the expulsion of sperm and, if it was white and viscous, the fish were considered to be ready for breeding. A total of 45 single pair matings were conducted in separate 7–10m^3^ hatching tanks.

Spawning usually occurred about 36 h after hormone admission. During this period, water temperature and salinity in the spawning tanks were maintained at 28.5–30.5°C and 32.0–33.0 ppt, respectively. Twelve hours after spawning, the fertilized (floating) eggs were collected and transferred to 500 L incubation tanks for hatching (each family in a different tank). The collection date of each egg was recorded.

### Nursing, rearing, and grow-out testing

From day 2 until day 15, the larvae were fed rotifer (*Brachionus plicatilis*). *Artemia nauplii* were introduced at day 10 and fed until day 30. Subsequently a commerical pellet feed (composition 55% protein; 9% lipid, <9% fiber, <8% moisture) was used when larvae was about 23–25 days. During the larval rearing water temperature ranged from 25.2 to 29.4°C (yearly average water temperatures were 27.3°C in the morning and 28.2°C in the afternoon). Salinity was 33.4 g L-1 (range 31.4–34.5 g L^−1^), pH 8.2 (range 7.9–8.4), DO >5.1 mg/L (range 4.7–5.4 mg L^−1^), and Secchi clarity > 1.8 m. When the fish reached a weight of 28 ± 18 g (Length = 13 ± 2 cm), 60 indviduals per family were marked using Passive Intergrated Transponder (PIT) tags for indvidual identifcation. After tagging, fish of all the families were pooled and conditioned in large size tanks for 1 week. The survival rate from hatching to 30 days averaged 38.4%, but from day 30 to fingerling stage (about 60 days) it was high (average 91%). Communal grow-out of all families was conducted in 24 seacages (4 × 4 × 2.5 m) with similar number of fish per family in each cage (200 fish per cage). Once the fish were transferred to the sea cage system, they were fed a formulated diet for 2 weeks and then trash fish twice daily at a feeding rate of ~10% body weight in the first 2 months (~200 g per fish), 5% of body weight when fish weight ranged from 200 to 500 g and 3% of body weight when weight exceeded 500 g per fish. In addition to sea cages, representatives of each family were tested in 200 m^3^ tank (water depth of 1.5 m) at an initial stocking density of 800 fish per tank. Only commercial pellet feed containing 45% protein and 12% fat (Uni-President Ltd) was used in tanks. Water quality was monitored once a week.

During the grow-out period of 17–18 months, fish were measured six times (one measurement every 3–4 months). One to 2 days prior to the measurements, the fish were conditioned in cages without food. Three main traits were recorded: growth related characteristics (body weight, total length), survival, and sexual maturity. A detailed description of the measurement methods is given in sections Family Production in Subsequent Generations in 2014/15 and 2017 and Other Measurements. The individual tag number, deformity and health conditions were also recorded. Digital scale and ruler were used for measuring weight and length. Survival was recoded as a binary trait and coded as 1 if the fish were still alive as 0 if the fish were absent at the final measurement and this trait was also expressed as percent difference in the number of fish at stocking and final harvest (570 dph).

### Other measurements

#### Maturity

Maturity of females was determined by biopsy as described above (section Family Production in Subsequent Generations in 2014/15 and 2017). The maturity of males was determined by stripping method. They were recorded in the form of presence and absence (coded as 1 and 0, respectively).

#### Deformity

Morphological deformity included a range of measures, namely lower jaw, nasal erosion, abnormal skeleton and deformed operculum (Nguyen et al., 2016, 2018a).

#### Food conversion ratio (FCR)

Total amount of feed provided in each net cage consumed by the fish was recorded to calculate FCR. This trait was not included in analysis because it was not possible to collect the amount of uneaten feed in sea cages.

### Traits used for genetic analysis

Due to the limited data records and details for sexual maturity, deformity and health condition, they were not included in our genetic analysis. This study focussed on three main traits: body weight, total length and survival. Body weight and length were measured at six different time points: 105, 180, 270, 360, 450, and 570 dph. These body traits showed continuous variation and were analyzed using linear mixed model. However, survival data was treated as a binary trait and analyzed using generalized mixed model as below (section Statistical Analysis).

### Statistical analysis

#### Linear mixed model

Heritability (the observed/measurable variations that are due to genetic inheritance) for traits showing continuous expressions (body weight and length) was estimated using Restricted Maximum Likelihood Method (REML) in a uni- or multi-variate mixed model (Henderson, 1975). Numerator relationship matrix was calculated from the pedigree that included both full- and half-sib families. The number of half-sib families in generations 2015 and 2017 was 12 and 22, respectively. In mathematical notation, the mixed model is written as:

(1) $$\begin{array}{r} y_{ijklm}=\mu +Y_{\text{i}}+E_{\text{j}}+s_{\text{k}}+d_{\text{l}}+e_{ijklm} \end{array}$$

where *y*_*ijklm*_ is the vector of observations for traits studied, *Y*_*i*_ is the systematic fixed effect of spawning year (2011, 2013, 2015, and 2017), and *E*_*j*_ is the fixed effect of testing environments. The non-significant effect of sex (*P* > 005) was omitted from the final model. The random factors in the model were sire (*s*_*k*_) and dam (*d*_*l*_). The dam component ($\sigma_{D}^{2}$) is most likely a combination of maternal and common environmental effects (thus,$\sigma_{D}^{2}=\sigma_{M+CE}^{2}$ , referred to as $\sigma_{C}^{2}$) caused by the separate rearing of full-sib families until individuals reached a suitable size for physical tagging. The log likelihood ratio test (LTR) showed that the common full-sib effect was significant (*P* < 0.05). The term *e*_*ijklm*_ signifies residual errors.

Heritabilities for body traits were estimated from a univariate model (Equation 1). The genetic variance ($\sigma_{A}^{2}$) was calculated as 4$\times \sigma_{S}^{2}$ where $\sigma_{S}^{2}$ is sire variance (Falconer and Mackay, 1996). The dam variance component ($\sigma_{D}^{2}$), in this case, was a combination of the maternal, dominant and common environmental effects, also named as common full-sib effects ($\sigma_{D}^{2}=\sigma_{C}^{2}$). The “and(dam)” option used in ASReml assumed equal sire and dam variances ($\sigma_{S}^{2}=\sigma_{D}^{2}$)(Ninh et al., 2014). The phenotypic variance ($\sigma_{P}^{2}$) was calculated as the sum of the additive genetic sire variance

($\sigma_{S}^{2}$), the dam variance ($\sigma_{D}^{2}$), the common full-sib ($\sigma_{C}^{2}$) and the residual variance ($\sigma_{e}^{2}$), as $\sigma_{P}^{2}=\sigma_{S}^{2}+\sigma_{D}^{2}+\sigma_{C}^{2}+\sigma_{e}^{2}$ or $\sigma_{P}^{2}=2\sigma_{S}^{2}+\sigma_{C}^{2}+\sigma_{e}^{2}$. The heritability was calculated as $h^{2}=\frac{\sigma_{A}^{2}}{\sigma_{P}^{2}}$ and the common environmental effect was calculated as $c^{2}=\frac{\sigma_{C}^{2}}{\sigma_{P}^{2}}$. Genetic and phenotypic correlations were estimated from a two-trait sire and dam model with the same fixed effects as shown in Equation (1). The correlations were calculated as the covariance divided by the product of the standard deviations of traits: $r=\frac{\sigma_{12}}{\sqrt{\sigma_{1}^{2}}\sqrt{\sigma_{2}^{2}}}$ where $\sigma_{12}$ was the estimated additive genetic or phenotypic covariance between the two traits, and $\sigma_{1}^{2}$ and $\sigma_{2}^{2}$ are the additive genetic or phenotypic variances of traits 1 and 2, respectively.

To examine the interaction between genotype and environment (G × E), the expressions in tank and cage were treated as different traits. A multi-trait model was applied to estimate the between-environment genetic correlations through a numerator relationship matrix obtained from the pedigree. In this analysis, Model 1 was used and all the effects were the same as shown in Equation (1), except that the effect of environment was excluded. Due to differences in standard deviations between the two culture systems, body weight, and length were square root transformed to estimate the between-environment genetic correlations. As the phenotypes were measured on different animals in tank and pond, there is no phenotypic correlation between trait expressions between the two environments.

#### Generalised threshold model

In this study, survival was treated as a binary character (0 = dead/or missing and 1 = alive). In addition to the linear mixed model (1), this trait was also analyzed using a threshold sire and dam model. The threshold models assume that the data follow a binominal distribution and logit (model 2) and probit (model 3) functions were used.

With the threshold logistic sire (*s*_*m*_) and dam model (2), heritability for survival was calculated using the variance of the logit link function, which implies a correction of the residual variance by factor π^2^/3.

$$\begin{array}{l} h^{2}=\frac{4\sigma_{s}^{2}}{\sigma_{s}^{2}+\sigma_{d}^{2}+\sigma_{e}^{2}\frac{\pi^{2}}{3}} \end{array}$$

where $\sigma_{s}^{2}$ is sire variance, $\sigma_{d}^{2}$ is dam variance, and $\sigma_{e}^{2}=1$

Under model 3 (probit threshold model), the heritability for survival was calculated as:

$$\begin{array}{l} h^{2}=\frac{4\sigma_{s}^{2}}{\sigma_{s}^{2}+\sigma_{d}^{2}+\sigma_{e}^{2}} \end{array}$$

where $\sigma_{s}^{2}$ is sire variance, $\sigma_{d}^{2}$ is dam variance, and $\sigma_{e}^{2}=1$

For binomial observations, estimates of *h*^2^ on the observed scale (0/1) were transformed to the liability scales (logit and probit) using the formula of Robertson and Lerner (1949):

$$\begin{array}{l} h_{L}^{2}=\frac{h_{O}^{2}p\left(1-p\right)}{z^{2}} \end{array}$$

where $h_{O}^{2}$ is the heritability on the observed (0/1) scale, $h_{L}^{2}$ is the estimated heritability on the liability (logit or probit) scale, *p* is a proportion of a given survival rate in the data, and *z* is the height of the ordinate of normal distribution corresponding to a truncation point applied to *p* proportion of survival.

The REML and mixed model approach have been implemented in ASReml version 4.0 (Gilmour et al., 2009). ASReml provides flexibility to specify different co-variance structures or different fixed and random effects to avoid any possible bias associated with the genetic parameter estimates.

## Results

### Basic statistics and characteristics of the data

The body weight and length measured six times during the grow-out phase from 105 to 570 days post-hatch (dph) are given in Table 1. The weight and length of the fish increased steadily until 270 dph after which there was a rapid increase in growth rate until the final harvest. At 570 days, the mean body weight was 2.3 kg and mean length was 53.9 cm. The coefficients of variation for body traits were greater in the earlier (105, 180, and 270 dph) than later phase of growth development (360, 450, and 570 dph). The growth curve of the experimental fish at six different grow-out periods is presented in Supplementary Figure 1. Pedigree structure of the data is shown in Table 2.

**Table 1:** Number of observations (n), mean, standard deviation (SD), coefficient of variation (CV, %), minimum, and maximum values for body weight and length during the growth trajectory.

**Trait**	**Unit**	**n**	**Mean**	**SD**	**CV**	**Min**	**Max**
W1	g	3,810	28.5	18.0	63.1	7.8	100
W2	g	3,488	211.0	86.7	41.3	15	600
W3	g	2,042	492.8	150.9	30.6	150	1,315
W4	g	2,537	1,073.0	131.3	12.2	570	1,725
W5	g	529	1,624.2	83.5	21.0	780	2,020
W6	g	1,151	2,247.8	196.2	8.7	1,580	3,500

L1	cm	3,815	13.2	2.0	15.4	7.4	25
L2	cm	3,477	24.8	5.2	21.1	16	100
L3	cm	2,042	32.5	3.3	10.2	21	42
L4	cm	2,535	43.1	1.8	4.8	34	49
L5	cm	5,29	49.6	1.5	8.7	48	57
L6	cm	1,151	53.9	1.7	3.1	48	63

Survival	%	2,649	48.1	49.9	103.8	0	1

*W1, W2, W3, W4, W5 and W6 = Body weight at 105, 180, 270, 360, 450 and 570 days post-hatch (dph). L1, L2, L3, L4, L5 and L6 = Total length at 105, 180, 270, 360, 450, and 570 days post-hatch, respectively*.

**Table 2:** Number of progeny, sire, and dam in spawning years.

**Year**	**Population**	**Progeny**	**Sire**	**Dam**
2011	Founder	369	–	–
2013	Base population	900	30	30
2015	Breeding	1380	41	43
2017	Breeding	1918	55	55
Total		4567	126	128

2015: 160 individuals from 16 full-sib families (15 sires and 16 dams) reared in tank

*2017: 611 individuals from 55 full-sib families reared in tank*.

**Figure 1:**
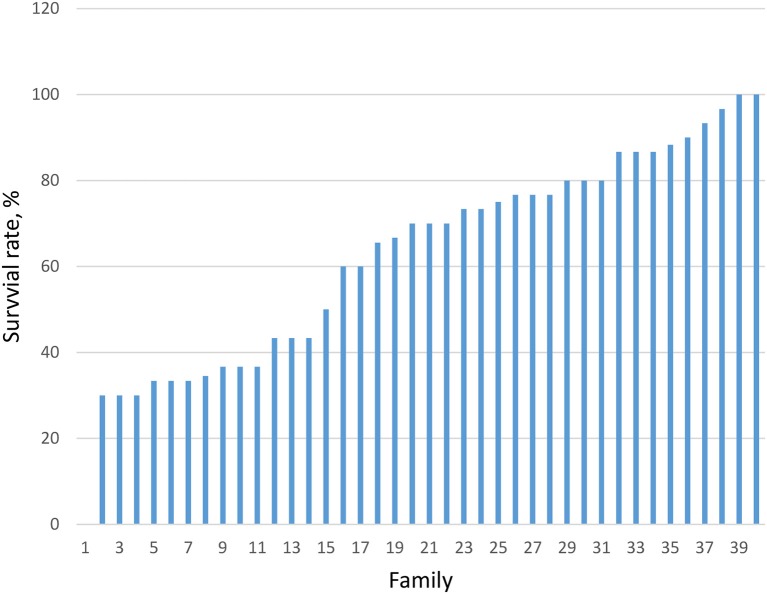
Variation in survival rate among 40 families produced in 2015 (*P* < 0.001).

Survival trait during grow-out phase (105 to 360 days) ranged from 30 to 100% among 30 families produced in 2013 and 40 families in 2015 (averaging 48.1%). Significant (*P* < 0.05) differences in the survival rates among the families in 2015 is presented in Figure 1.

### Effects of culture environments

**Figure 2:**
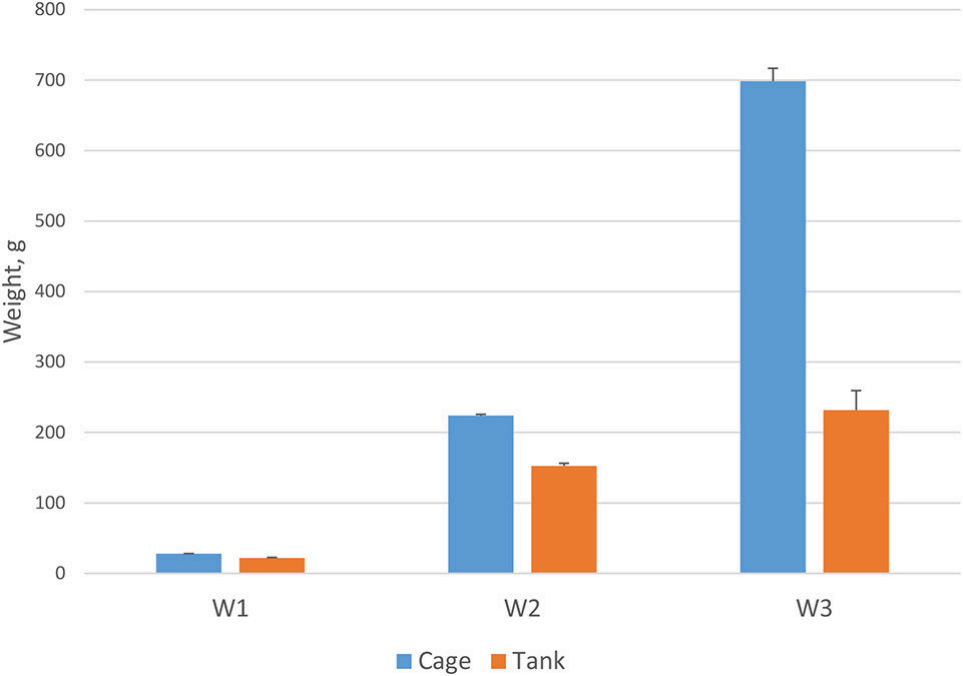
Fish body weight in cage (*n* = 2058) and tank (*n* = 576) over different measurement periods at 105, 220, and 270 days post-hatch (*P* < 0.001).

A prevailing commercial production system for Asian seabass is sea cage, but tank culture is also increasingly practiced. In this study, growth traits were investigated in two different culture systems: tanks and sea cages. The fish grew significantly faster (*P* < 0.001) in sea cages than in tanks (Figure 2). After 270 days, average weight of fish stocked in sea cages was 698.5 g (373 mm length) and in tanks 231.6 g (291 mm length). The differences in weight and length between the two environments varied during the growing period and were significantly (*p* < 0.001) greater after 270 dph than during the early phase of growth (66% for W3 vs. 21% for W1).

### Sex differences in growth traits

**Figure 3:**
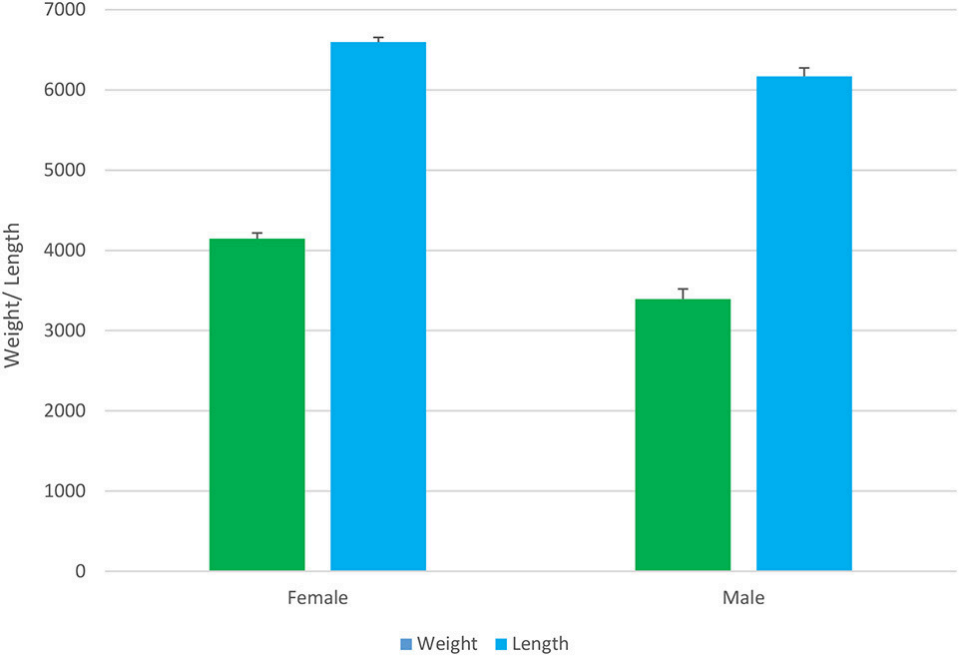
Body weight (g) and length (cm × 100) of female and male barramundi (*P* < 0.001).

Between-sex differences in growth was examined in parental candidates prior to breeding. In this population of Asian seabass, the females had significantly (*P* < 0.001) greater body weight than the males (4.2 vs. 3.4 kg; Figure 3). The sexual differences in body weight were not significant during the grow-out phase either in tanks or cages. However, the between-sex differences in growth-related traits are widely observed in aquaculture, such as tilapia and prawn (Hung et al., 2014).

### Heritability and common full-sib effects for growth traits

The estimated heritabilities (h^2^) for body weight and length at different times of measurement were moderate to high (Table 3). The heritability estimates ranged from 0.12 to 0.78 for body weight and 0.41–0.85 for total length. Except for W5 (h^2^ = 0.12) that was likely due to measurement errors, the heritability for both traits was higher at 105 to 450 dph of on-grown than at other measurement periods. Across all measurements, the heritability estimates for weight and length were significant.

**Table 3:** Heritability (h^2^) and common full-sibs (c^2^) for traits studied.

**Traits**	**h^2^**	**c^2^**
W1	0.78 ± 0.12	0.22 ± 0.04
W2	0.43 ± 0.12	0.19 ± 0.04
W3	0.48 ± 0.14	0.12 ± 0.04
W4	0.61 ± 0.13	0.07 ± 0.03
W5	0.12 ± 0.13	0.08 ± 0.04
W6	0.75 ± 0.21	0.03 ± 0.05

L1	0.70 ± 0.11	0.18 ± 0.04
L2	0.45 ± 0.09	0.05 ± 0.02
L3	0.62 ± 0.15	0.15 ± 0.05
L4	0.65 ± 0.14	0.10 ± 0.04
L5	0.85 ± 0.18	0.02 ± 0.03
L6	0.41 ± 0.28	0.05 ± 0.07

Survival^1^	0.16 ± 0.04	
Survival^2^	0.19 ± 0.18	0.08 ± 0.05
Survival^3^	0.21 ± 0.19	0.08 ± 0.05

Survival^1^ = Linear mixed, Survival^2^ = Threshold logistic model and Survival^3^ = Threshold probit model

The common full-sib (c^2^) effects accounted from 2 to 22% of the total variation for weight and length. The c^2^ effects were larger for W1-W3 than those obtained for W4-W6 (16–22% vs. 2–10% for body weight).

### Heritability estimates for survival using different statistical models

Three models were used to estimate heritability for survival. The heritability estimate from linear mixed model (model 1) was $h_{o}^{2}$ = 0.05. Threshold probit model (model 3) gave the highest value at $h_{L}^{2}$ = 0.21, whereas the estimate obtained from threshold logistic model (model 2) was intermediate ($h_{L}^{2}=$ 0.19). When the observed heritability obtained from the linear mixed model (1) was back-transformed to the underlying liability scale, the estimates were generally similar between the linear and threshold models (2 and 3). However, across the three statistical models used, the heritabilities for survival on both observed scale (model 1) and underlying liability (models 2 and 3) were not different from zero due to their high standard errors (Table 3). The common full-sib effect on survival was low (8%).

### Phenotypic and genetic correlations

Phenotypic and genetic correlation of body weight between different times points are presented in Table 4. In general, genetic correlation of weight traits estimated for four measurements were all positive (0.31 to 0.62) and significantly differed from one. The genetic correlation (*r*_*g*_) between weight and length at the same age was high (0.95 to 0.99), except for the moderate estimate between W2 and L2 (*r*_*g*_ = 0.38). However, the *r*_*g*_ values between weight and length at different ages were low or moderate, of which two estimates between W1 and L4 or between W4 and L2 were not different from zero, due to their high standard errors. Overall, the genetic correlations of traits between successive rearing periods were stronger than those that are further apart. The phenotypic correlations were all positive and significant.

**Table 4:** Phenotypic (above) and genetic (below the diagonal) correlations.

**Traits**	**W1**	**W2**	**W3**	**W4**	**L1**	**L2**	**L3**	**L4**
W1		0.47 ± 0.03	0.40 ± 0.03	0.25 ± 0.03	0.87 ± 0.01	0.29 ± 0.03	0.39 ± 0.05	0.38 ± 0.05
W2	0.47 ± 0.08		0.63 ± 0.02	0.47 ± 0.02	0.50 ± 0.03	0.44 ± 0.02	0.65 ± 0.02	0.49 ± 0.03
W3	0.56 ± 0.07	0.62 ± 0.07		0.43 ± 0.03	0.39 ± 0.04	0.35 ± 0.03	0.90 ± 0.01	0.46 ± 0.04
W4	0.31 ± 0.09	0.49 ± 0.08	0.44 ± 0.11		0.42 ± 0.04	0.19 ± 0.03	0.38 ± 0.04	0.84 ± 0.01
L1	0.96 ± 0.01	0.78 ± 0.09	0.63 ± 0.14	0.84 ± 0.07		0.16 ± 0.03	0.40 ± 0.03	0.27 ± 0.03
L2	0.41 ± 0.15	0.38 ± 0.16	0.28 ± 0.17	−0.11 ± 0.19	0.30 ± 0.10		0.33 ± 0.02	0.19 ± 0.03
L3	0.55 ± 0.15	0.69 ± 0.13	0.99 ± 0.01	0.19 ± 0.22	0.50 ± 0.08	0.56 ± 0.09		0.42 ± 0.04
L4	0.06 ± 0.12	0.42 ± 0.17	0.11 ± 0.23	0.95 ± 0.02	0.41 ± 0.09	0.26 ± 0.10	0.33 ± 0.12	

### Genotype by environment (G×E) interaction

Table 5 shows the between-environment genetic correlations (*r*_*g*_) for weight traits. Due to the limited data records at 450 and 570 dph in both cages and tanks, the between-environment genetic correlations were estimated for body weights at three ages: 180 (W2), 270 (W3), and 360 dph (W4). The genetic correlation between tank and cage culture was high (0.91–0.94) for W2 and W3. The genetic correlation estimate (0.84 ± 0.12) was similar for total length. However, the between-environment genetic correlation decreased as growth progressed (*r*_*g*_ = 0.59 ± 0.33 for W4, 360 dph). Our results suggest that the effect of G × E interaction is potentially important for growth traits measured in later stages of development in Asian seabass. This is particularly significant when the commercial production system (e.g., tank) is incompatible with a controlled maintenance of broodstock in the selection nucleus (e.g., cage).

**Table 5:** Effect of genotype by environment (G × E) interaction.

**Traits**	**Between-environment genetic correlation**
W2	0.91 ± 0.10
W3	0.94 ± 0.12
W4	0.59 ± 0.33

## Discussion

### Heritability for survival

The heritability estimate for survival during the grow out phase from 105 days post-hatch (dph) to 570 dph was low (<12%) in this study. This is as expected for binary characters that often require a larger sample size to obtain reliable heritability estimates (Davies et al., 2015; Nguyen et al., 2018b). Further, survival as well as fitness-related traits are known to be influenced by many environmental factors, such as nutrition, feeding regimes, stocking density, or social competition (Thoa et al., 2016). This is also because fitness-related traits exhibit small additive genetic components and thus, heritabilities are low for these characters. Despite the low and non-significance of the heritability estimates, our study showed that there are heritable genetic components determining survival during grow-out. This is supported by the highly significant differences in survival rate among families studied (Figure 1). Our results are consistent with observations in other aquaculture species which show that the heritability for survival traits during grow-out phase was low (0.05–0.17), such as tilapia (Hamzah et al., 2017a), common carp (Dong et al., 2015), rainbow trout (Vehviläinen et al., 2012) and giant freshwater prawn (Vu et al., 2017). Some other studies, however, reported moderate to high heritability (h^2^ = 0.53) for the survival trait (Ninh et al., 2014). A meta-analysis of 31 studies across aquaculture species (Nguyen, submitted) showed that the weighted mean heritability for survival rate was 0.14 ± 0.03, suggesting that improving this character by selection, although possible, may be slow due to environmental factors.

### Heritability for growth traits

There is limited information regarding the additive genetic variability for growth trajectory in Asian seabass. In this study, we report, for the first time, the heritability for growth traits at six different time points (105–570 dph) in a selected population of Asian seabass. The moderate to high heritabilities obtained for body weight and length across six growth stages indicate that there is substantial genetic variation in growth related traits (weight and length). This suggests that selection for growth should be effective in this population. By contrast, previous research reported heritability for juvenile fish 90 dph (≈18 g or ≈10 cm in length). The published h^2^ estimates ranged from 0.12 to 0.24 for weight, length and condition factors (Wang et al., 2008b) or 0.15 to 0.22 in 62 dph fish (Domingos et al., 2013). These h^2^ estimates generally had high standard errors (0.09 to 0.22) and may have been overestimated because the common full-sib effects were not accounted for in statistical models. The significant heritability values obtained from our study indicate that selection to improve body traits should be effective in this population. The heritability reported for growth traits were moderate to high in other species, such as kingfish (Premachandra et al., 2017), salmon or giant freshwater prawn (Hung and Nguyen, 2014).

### Common full-sib effects

The common full-sib effects (c^2^) observed in the present study was due to separate rearing of each family in different tanks before tagging. The c^2^ effect accounted for between 3 and 22% of the total variation for weight and length. Our c^2^ estimate is in good agreement with those reported in other marine (Falica et al., 2017) and freshwater (Hamzah et al., 2017b) fish species. The magnitude of the c^2^ effect generally diminished with the growth time (Table 3) and the c^2^ estimate was significantly larger for growth traits during the early phase of growth development than those at harvest (Oliveira et al., 2016). Strategies to reduce the c^2^ effect in selective breeding programs can include: (i) shortening the spawning and nursing time for full- and half-sib families; (ii) physical tagging of the fingerlings at an early age as possible; or (iii) early communal rearing after birth using molecular techniques for parentage assignment. In breeding programs where these factors can be well managed, the c^2^ effects were small and there may be little improvement by including them in the evaluation of breeders (Winkelman and Peterson, 1994; Martinez et al., 1999; Pante et al., 2002; Gjerde et al., 2004; Kjøglum et al., 2005). For this barramundi population, inclusion of the c^2^ effect in statistical models is deemed essential in order to minimize possible biases in genetic parameter estimates and predicted breeding values that may lead to reductions in selection accuracy and hence, a reduction in genetic gain.

### Correlations at different measurements

Genetic correlations of body weight between different time points are all positive. However, the estimates differed significantly from one, suggesting that body traits at different growth phases in this population are genetically different. Hence, selection in the early stage of growth development may not capture all genetic expression at later stages of growth or at the time of commercial harvest. This contrasts with results reported in other aquaculture species. For instance, Ninh et al. (2013) showed high genetic correlations (0.85–0.99) for body weight at 3, 7, and 12 months of age in common carp. A similar correlation was also found in giant freshwater prawn (Hung et al., 2014).

Consistent with the results reported for other species, the genetic correlations between weight and length measured at the same time/age were high (0.88–0.99) in this population of barramundi. The genetic correlation between weight and length in our study were close to one as were those reported in Atlantic salmon and rainbow trout (Gjerde and Gjedrem, 1984), tilapia (Nguyen et al., 2010), carp (Ninh et al., 2011), or shrimp (Nguyen et al., 2014). This suggests that these growth traits are under control by a similar or same set of genes. Our results indicate that either body weight or length can be used as selection criterion in aquaculture species. However, measurement of length at an early age may not be a good predictor of body weight at other growth periods because in our research the genetic correlations between these traits differed significantly from one.

### Genotype by environment (G×E) interaction

Multi-variate assessment of genotype by environment (G × E) interaction suggests that the G × E effect was not important in the first year of on-growth; however, it had significant impact on body traits in adult fish. The longer the fish were grown, the stronger the G × E effects had on body traits. Thus, selection of breeding candidates in sea cages may not be effective when the fish are cultured in tanks. However, in a study with barramundi, Domingos et al. (2013) reported close to one genetic correlations (0.87–0.99) for homologous body traits between fresh and sea water (62 dph) and between tank (343 dph) and pond (469 dph) environments. G × E interactions between tank and cage have not been reported for traits of economic importance in adult barramundi and no comparison is possible with the results obtained from the present study. During sea cage culture, a range of environmental factors may be influential including: ambient temperature, water parameters, feeds and feeding, culture system, and management and husbandry practices. For example, Asian seabass brooders raised in a sea cage net had better growth performance than those raised in tanks (Wang et al., 2008b) and those reared in intensive tanks outperformed their family counterparts in a semi-intensive pond (Domingos et al., 2013). Our results also showed that the fish grew faster in sea cages than in tanks.

The G × E interaction effects have been examined in many aquaculture species (Nguyen, 2016; Sae-Lim et al., 2016) and have shown that, when the environments are “similar” (e.g., freshwater pond *vs*. freshwater cage), the G × E effects may not be important for growth traits. However, when the selection and production environments differed greatly (e.g., sea cages vs. freshwater tanks) the G × E is of biological significance. G × E effects resulted in a reduced genetic gain and lowered genetic parameter estimates in red tilapia (Nguyen et al., 2017). In these cases, the G × E interaction effects must be accounted for in genetic improvement programs or, when the economic losses are greater than the cost of running a new breeding program, separate genetic lines should be developed for each culture environment. Continuing accumulation of the growth data in future generations would enable the better assessment of the G × E effects in this population of barramundi.

### Experience and challenges

Despite some initial achievements in this study (including a new set of genetic parameters and identifying the G × E interaction effects for growth traits), practical implementation of the breeding program for Asian seabass is faced with several challenges. The biggest problem is achieving synchronized spawning of mating pairs in order to produce many families within a reasonable time interval (2–3 weeks). In the population we studied, the fish generally reached maturity after two years of age although at this stage the sex ratio was uneven with a much larger proportion of males (43.1%) than females (8.2%). The proportion of fish whose sex was not identified was 48.7%. Furthermore, females after harvest (average 2.3 kg) were not ready to spawn and the breeding failure rate was high. In the base population (G0), parental fish were injected using LHRHa and kept in tanks to allow natural spawning. With this method, the spawning rate was only 66%. Since G1, artificial insemination (i.e., stripping of eggs and collection of sperm after HCG admission) increased spawning success to 78% although fertilization and hatching rates were low (53%) compared with natural spawning in tanks (66%). Due to these problems, selection had to be made by using lower EBV candidates and thus, affecting genetic parameters and genetic progress achieved for growth traits in this population. In addition, larval and post-larval rearing showed unwanted cannibalism. To overcome this problem, “shooters”/or cannibals (unusual large size fish) were removed from rearing tanks twice a week. Grading was not used to minimize possible bias in genetic parameter estimates. As a result, fingerling weight/size varied greatly among families studied and affected growth performance in both tanks and sea cages. The common full-sib effects were also important for body traits (2–22% of total variance) in this population. Changing environments in some years had noticeable negative impacts on the growth and health of the breeding population as a result of parasitic and bacterial diseases. Main diseases included the protozoal disease Trichodiniasis, fish louse due to a parasitic crustacean, and a bacterial disease Vibrosis and generally caused reductions in growth and maturation rates in on-grow fish and breeding candidates. In 2013, significant loss (70% of parental candidates) occurred when the water temperature dropped below 15°C during the season before winter. Further, operculum deformity (opaque eyes) due to parasites or environmental factors occurred in 60% of the on-grow fish even though a freshwater bath using KMnO_4_ was applied once a week. Obviously, morphological deformity and parasite diseases (e.g., skin fluke) are crucial to Asian seabass and should be recorded and included in genetic analyses. A fourth problem was that in the first 2 years of the breeding program there were not enough spawning/rearing tanks to accommodate all the families produced for subsequent performance testing in sea cages and tanks. Small size of sea cages also affected growth rate of the experimental fish. Finally, live foods, such as scad or other trash fish, were not always available and this affected the spawning rate and reproductive performance of brood fish. Alternative diets are sought to overcome these problems. As a consequence, the number of families expected to produce in each generation (at least 60) was not always achieved. Mass spawning together with parentage assignment techniques using DNA markers to enable early communal rearing of all families soon after birth may be an option to be considered in the future breeding program for Asian seabass.

## Conclusions

There are heritable genetic variations for growth traits that can be exploited by genetic selection in selective breeding programs for barramundi. However, selection for growth in the early stages (e.g., 105, 180, or 270 dph) may not capture all the genetic variation necessary in later rearing periods. This is because genetic correlations of body weight at different ages, although positive and moderate, differed significantly from one. The genetic correlation for body weight expressions between tank and sea cage culture decreased as growth progressed especially after 360 dph, suggesting that the relationship between genotype and environment is important for body traits including weight and length in this population of Asian seabass. In conclusion, selection for growth traits should be made at (or close to) harvest to maximize commercial production. Continuing collection of growth data in different environments is needed to ascertain the G × E interaction effect and if it is significant there may be a call to have separate breeding programs for tank and cage environments. Expansion of the breeding objectives by including new traits, namely improved disease resistance and reduced cannibalism would maximize productivity and revenue for Asian seabass aquaculture.

## Author contributions

PK, TP, ND, WK, and NN conceived and designed the experiments, analyzed the data, and prepared and approved the manuscripts.

### Conflict of interest statement

The authors declare that the research was conducted in the absence of any commercial or financial relationships that could be construed as a potential conflict of interest.
