# Supplementary material for: An 8-Year Breeding Program for Asian Seabass Lates calcarifer: Genetic Evaluation, Experiences, and Challenges
Source: Front Genet. 2018 May 29;9:191. doi: 10.3389/fgene.2018.00191 (PMC5987403; doi:10.3389/fgene.2018.00191)
Supplement: Supplementary file 1 [file Table_1.docx]

**Supplementary Table S1**: Populations and locations of eight collected stocks

| Populations | Locations (*) | Fish number | Average length (cm) | Average weight (g) |
| --- | --- | --- | --- | --- |
| Wild: Hai Phong | 20076’N - 107005’E | 382 | 10.3±0.2 | 12.4±0.6 |
| Hatchery: Hai Phong | 20^0^76’ N - 107^0^04’ E | 360 | 10.6 ±0.1 | 12.2±0.2 |
| Wild: Khanh Hoa | 12^0^20’ N - 109^0^19’ E | 388 | 10.5±0.3 | 12.6±0.2 |
| Hatchery: Khanh Hoa | 12^0^16’ N - 109^0^19’ E | 380 | 10.7±0.3 | 12.4±0.3 |
| Wild: Vung Tau | 10^0^40’ N - 107^0^08’ E | 364 | 10.6±0.2 | 12.6±0.2 |
| Hatchery: Vung Tau | 10^0^41’ N - 107^0^13’ E | 360 | 10.4±0.1 | 12.2±0.4 |
| Wild: Kien Giang | 9^0^66’ N - 104^0^40’ E | 385 | 10.6±0.2 | 12.4±0.2 |
| Hatchery: Kien Giang | 90^0^66’ N - 104^0^30’ E | 420 | 10.7±0.3 | 12.6±0.3 |

(*) Locations represented area where the fingerlings were mainly collected.
