# Supplementary material for: An 8-Year Breeding Program for Asian Seabass Lates calcarifer: Genetic Evaluation, Experiences, and Challenges
Source: Front Genet. 2018 May 29;9:191. doi: 10.3389/fgene.2018.00191 (PMC5987403; doi:10.3389/fgene.2018.00191)
Supplement: Supplementary file 2 [file Table_2.docx]

**Supplementary Table S2:** Growth performance, survival, food conversion ratio, maturation of eight stocks in cage in 17-month period

Values are mean ± standard deviation (n=30); Means in the same row with the same superscript are not significantly different (p>0.05 from one other.

Wi = Initial weight, Wf= harvest weight, Li= Initial length, Lf = length at harvest, DWG = Daily weight gain, DLG = Daily length gain and FCR = Food conversion ratio

|  | Wild stocks | | | | Hatchery stocks | | | |  |
| --- | --- | --- | --- | --- | --- | --- | --- | --- | --- |
|  | Vung Tau | Khanh Hoa | Kien Giang | Hai Phong | Vung Tau | Khanh Hoa | Kien Giang | Hai Phong | |
| Wi (g) | 50.1 ± 0.2 | 50.3 ± 0.5 | 50.4 ± 0.2 | 50.1 ± 0.3 | 50.4 ± 0.5 | 50.1 ± 0.4 | 50.1 ± 0.1 | 50.1 ± 0.3 | |
| Wf (g) | 3300 ± 42^a^ | 3180 ± 64^a^ | 2990 ± 38^ab^ | 2968 ± 46^ab^ | 3154 ± 62^a^ | 2874 ± 42^b^ | 2678 ± 28^b^ | 2674 ± 34^b^ | |
| Li (cm) | 15.3±0.2 | 15.3±0.2 | 15.3±0.2 | 15.3±0.2 | 15.3±0.2 | 15.3±0.2 | 15.3±0.2 | 15.3±0.2 | |
| Lf (cm) | 61.0±0.9^a^ | 60.2±0.4^a^ | 59.2±0.2^ab^ | 59.0±0.5^ab^ | 59.6±0.3^a^ | 58.2±0.3^b^ | 57.8±0.6^b^ | 57.4±0.4^b^ | |
| DWG  (g day^-1^) | 6.378±0.382^a^ | 6.145±0.234^a^ | 5.776±0.352^b^ | 5.734±0.364^b^ | 6.095±0.334^a^ | 5.551±0.402^bc^ | 5.170±0.286^c^ | 5.163±0.224^c^ | |
| DLG  (cm day^-1^) | 0.089±0.004^a^ | 0.087± 0,001^a^ | 0.085±0.002^ab^ | 0,.085 ±0.003^ab^ | 0.086 ±0.002^a^ | 0.083 ±0.001^b^ | 0.083 ±0.001^b^ | 0,.082 ±0.001^b^ | |
| FCR | 3.90*±*0.22 | 4.03±0.08 | 4.53±0.14 | 4.64±0.10 | 3.98±0.12 | 4.76±0.12 | 4.50±0.10 | 4.99±0.16 | |
| Survival (%) | 87.9±1.4 | 85.8±0.8 | 90.8±0.2 | 85.8±0.4 | 90.8±0.6 | 85.8±0.4 | 91.7±0.6 | 90.8±0.2 | |
| Male maturation (%) | 56.9±0.8^a^ | 56.3±0.8^a^ | 39.4±1.6^b^ | 38.2±0.8^b^ | 54.1±1.2^a^ | 38.2±0.4^b^ | 31.5±0.8^c^ | 30.1±1.6^c^ | |
| Female maturation (%) | 13.3±0.8^a^ | 10.7±0.8^ab^ | 8.3±2.4^b^ | 5.5±0.4^c^ | 8.3±0.8^b^ | 7.8±0.8^b^ | 6.5±1.6^bc^ | 5.3±1.6^c^ | |
