# Supplementary figures and images for: An 8-Year Breeding Program for Asian Seabass Lates calcarifer: Genetic Evaluation, Experiences, and Challenges
Source: Front Genet. 2018 May 29;9:191. doi: 10.3389/fgene.2018.00191 (PMC5987403; doi:10.3389/fgene.2018.00191)

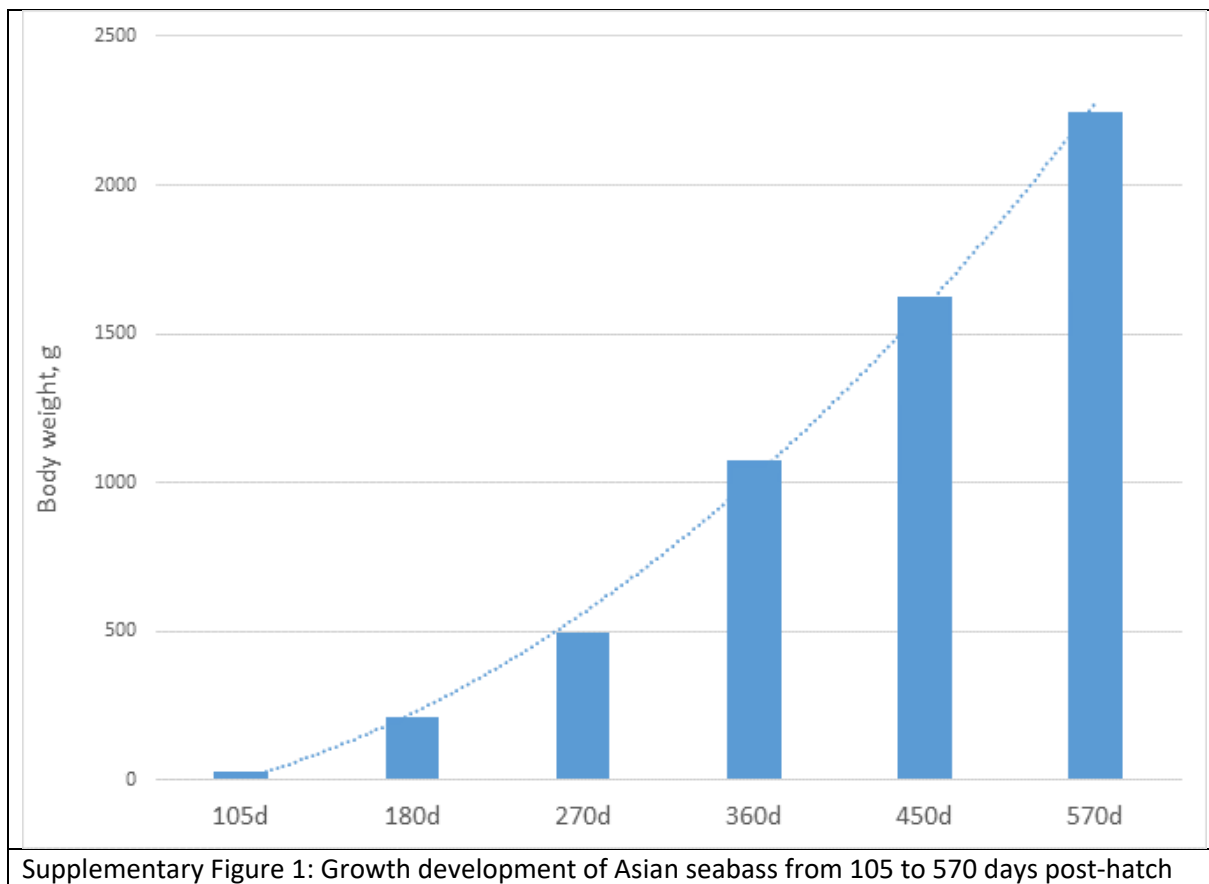

Supplement: Supplementary file 3 [file Image_1.pdf]
